# A plant virus attenuates the Toll immune pathway by degradation of Pellino to facilitate viral infection in insect vectors

**DOI:** 10.1128/jvi.00021-25

**Published:** 2025-03-31

**Authors:** Yu-Xiao Du, Yu-Hua Qi, Yan-Hua Lu, Bo-Xue Li, Yu-Juan He, Yan Zhang, Lin Lin, Chuan-Xi Zhang, Xiao-Wei Wang, Jian-Ping Chen, Gang Lu, Jun-Min Li

**Affiliations:** 1 State Key Laboratory for Quality and Safety of Agro-Products, Key Laboratory of Biotechnology in Plant Protection of MARA, Zhejiang Key Laboratory of Green Plant Protection, Institute of Plant Virology, Ningbo University 47862, Ningbo, China; 2 Yongjia County Agriculture and Rural Bureau, Yongjia, Zhejiang, China; 3 State Key Laboratory of Rice Biology, Ministry of Agriculture Key Lab of Molecular Biology of Crop Pathogens and Insects, Zhejiang Key Laboratory of Biology and Ecological Regulation of Crop Pathogens and Insects, Institute of Insect Sciences, Zhejiang University 12377, Hangzhou, China; Iowa State University, Ames, Iowa, USA

**Keywords:** plant virus, rice stripe virus, Toll immune pathway, antiviral factor, counter-defense, insect vector

## Abstract

Many plant viruses are persistently transmitted by insect vectors. The viral antagonism of insect innate immune responses is a critical step in ensuring persistent viral infection. Recent studies have shown that the Toll immune pathway mediates the persistent and propagative transmission of rice stripe virus (RSV) in its insect vector (*Laodelphax striatellus*). However, whether other host factors are involved in the Toll pathway and how RSV counteracts the Toll immune response in *L. striatellus* remain unclear. Here, we reported that LsPellino also inhibited RSV infection in *L. striatellus* by interacting with LsTube and participating in the Toll immune pathway. In contrast, the viral nonstructural protein NS3 hijacked the suppressor of cytokine signaling 5 (LsSOCS5) to promote the degradation of LsPellino via the 26S proteasome pathway, thereby suppressing the Toll immune response. In summary, these findings demonstrate that RSV attenuates the Toll immune pathway by degradation of LsPellino to facilitate viral infection in insect vectors. Our research provides new insights into controlling the transmission of vector-borne viruses.

**IMPORTANCE:**

Plant virus diseases pose a serious threat to global crop production. Nearly half of the known plant viruses are persistently transmitted by insect vectors, and these plant viruses must counteract various innate immune responses to maintain persistent infection. Here, we uncover a novel counter-defense mechanism against Toll antiviral defense. Our research showed that LsPellino exerts antiviral function by interacting with LsTube and participating in the Toll immune pathway. To counteract this immunity, a plant virus, rice stripe virus, attenuates the Toll immune pathway and promotes viral infection by using viral nonstructural protein NS3 to mediate the degradation of LsPellino in its insect vector, *Laodelphax striatellus*. This study not only contributes to a better understanding of the arms race between viruses and insect vectors but also provides a new perspective for controlling the transmission of plant viruses.

## INTRODUCTION

The Toll immune pathway is an evolutionarily conserved signaling pathway in both insects and mammals (1–3). When pathogens infect *Drosophila*, the receptor Toll binds to activated Spatzle (Spz), and three core components (MyD88, Tube, and Pelle) are recruited to form a receptor proximal oligomeric complex. This complex further triggers the degradation of Cactus and the phosphorylation of transcription factor Dorsal. Subsequently, the phosphorylated Dorsal translocates from the cytoplasm to the nucleus for regulating the expression of multiple antimicrobial peptides (4, 5). The Toll pathway has been reported to participate in various biological processes, including embryonic development and innate antiviral immunity (3, 6, 7). Besides, many host factors have been identified to be involved in this immune pathway, including the Pellino family (8–10).

Pellino proteins are a type of highly conserved E3 ubiquitin ligases and have been implicated in the regulation of PRR signaling pathway, including the Toll immune pathway (11). The mammalian Toll-like receptor (TLR) and interleukin-1 receptor (IL-1R) pathways are homologous to the *Drosophila* Toll pathway (12, 13). Pellino can interact with and be phosphorylated by IRAK4 (IL-1R-associated kinase 4), the vertebrate homolog of Tube (14–16). The mammalian Pellino family consists of three members (Pellino-1, Pellino-2, and Pellino-3), and only one Pellino is found in the fly (8). In mammals, Pellino-2 positively regulates TLR/IL-1R pathway and Pellino-3 negatively regulates IL-1R signaling (17, 18). Pellino-1 can both positively and negatively regulate TLR/IL-1R-dependent NF-κB activation (15, 19). In addition, *Drosophila* Pellino positively regulates innate immunity by interacting with the activated Pelle protein (9). Pellino has also been reported to target MyD88 for ubiquitination and degradation, acting as a negative regulator in the Toll signaling pathway in *Drosophila* (10). However, the role of the Pellino family in the transmission of plant viruses by insect vectors remains largely unknown.

Rice stripe virus (RSV) is a non-enveloped negative-sense RNA virus belonging to the genus *Tenuivirus* and family *Phenuiviridae*, causing severe losses to rice production in Asian countries (20). The genome of RSV contains four RNA segments and encodes one RNA-dependent RNA polymerase (RdRp), one nucleocapsid protein (NP), and five nonstructural proteins (NS2, NSvc2, NS3, NS4, and NSvc4) (21, 22). RSV is transmitted by the small brown planthopper (*Laodelphax striatellus*) in a persistent and propagative manner (23). When RSV enters the midgut lumen of *L. striatellus* through feeding on RSV-infected plants, RSV needs to overcome the midgut barrier and replicates in the epithelial cells. Subsequently, RSV spreads through the hemolymph system to the salivary glands and finally disseminates to healthy plants (24–27). In addition to replicating and proliferating in *L. striatellus*, RSV also invades the reproductive system and is vertically transmitted to the offspring (28–30). Recent studies have demonstrated that RSV infection activates several immune signaling pathways in *L. striatellus*, such as the small interfering RNA (siRNA) pathway (31), the c-Jun N-terminal kinase (JNK) pathway (32), the prophenoloxidase (PPO) pathway (33), and the Janus kinase-signal transducer and activator of transcription (JAK-STAT) pathway (34). Our previous studies showed that RSV infection also activates the Toll signaling pathway, which regulates downstream immune-related effectors to maintain persistent viral transmission (35, 36). Although Pellino has been reported to be involved in insect Toll antiviral immunity, it is still unknown whether Pellino affects the transmission of RSV by *L. striatellus*.

In this study, we aim to clarify the role of the Pellino family in the transmission of RSV by the small brown planthopper. We found that there was only one member of the Pellino family in *L. striatellus* (LsPellino). LsPellino exerted antiviral function by interacting with LsTube and participating in the Toll immune pathway. Meanwhile, RSV NS3 hijacked an E3 ubiquitin ligase, the suppressor of cytokine signaling 5 (LsSOCS5), to promote the degradation of LsPellino through the 26S proteasome pathway and suppress Toll immune response. Our results suggest that attenuation of Toll immune defense through RSV-mediated degradation of LsPellino is essential for persistent viral infection in insect vectors.

## RESULTS

### Identification and characterization of *LsPellino*

**Fig 1:**
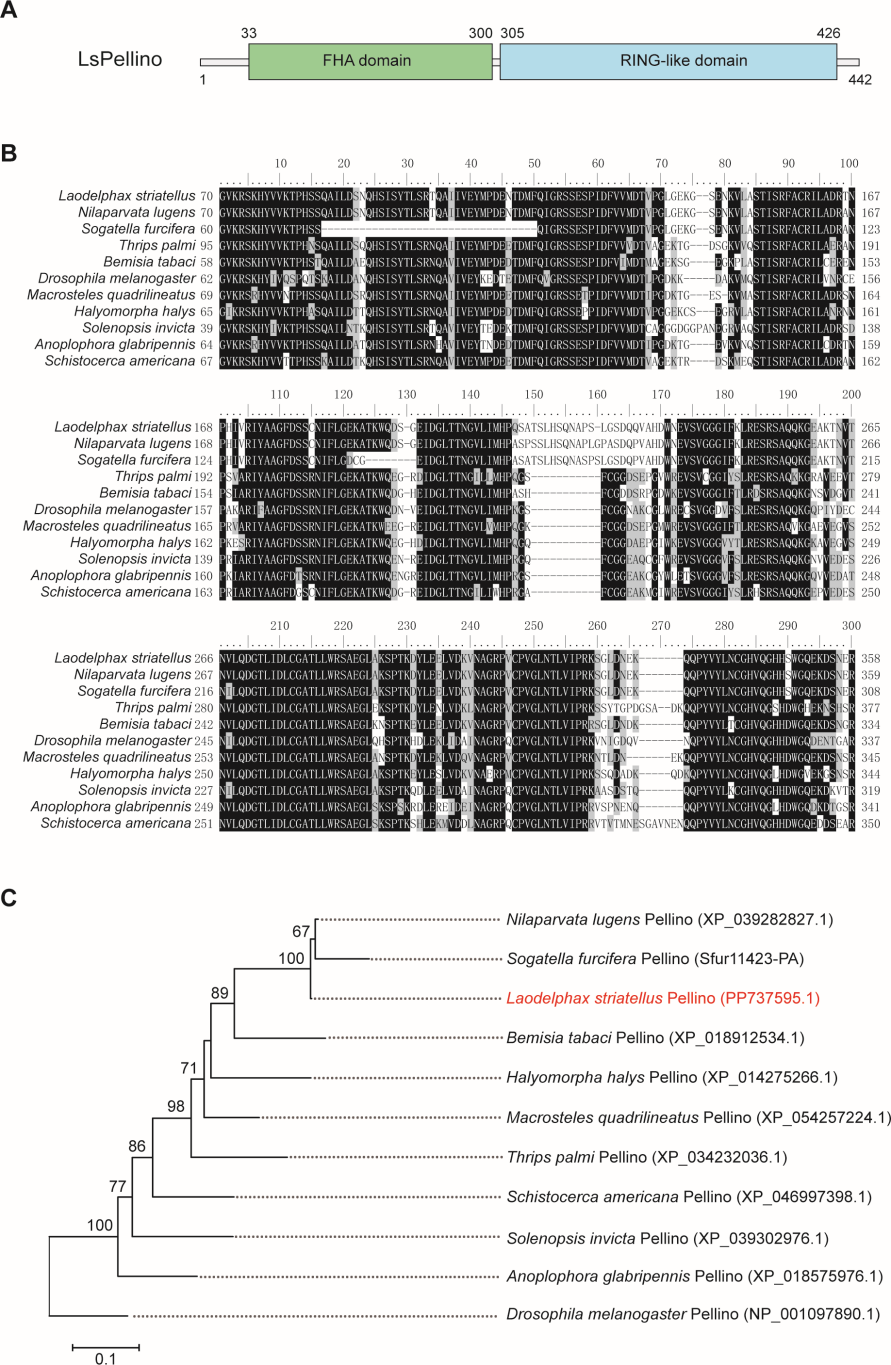
Structure feature and phylogenetic analysis of *L. striatellus* Pellino. (**A**) Schematic diagrams illustrating the FHA and RING-like domains of LsPellino. (**B**) Alignment of amino acid sequences of Pellino. Fully conserved amino acid residues are shown in black. Partially conserved amino acid residues are shown in gray. (**C**) Phylogenetic analysis of Pellinos from *L. striatellus* and other insect species. Pellinos from different species and their GenBank accession numbers are indicated on each branch. The LsPellino are marked with red font. Bootstrap values exceeding 60% are displayed at the corresponding nodes.

The *LsPellino* gene sequence was identified by comparing the genome of *L. striatellus* with *Pellino* genes from other insect species. The full sequence of *LsPellino* was confirmed by PCR amplification followed by Sanger sequencing. The open reading frame (ORF) of *LsPellino* included 1,329 bp and encoded a protein of 48.6 kDa with 442 amino acid (aa) residues. Conserved domain analysis showed that *LsPellino* contained two typical functional domains. The FHA (forkhead-associated) domain was predicted to extend from 33 to 300 aa, whereas the RING-like domain was predicted to extend from 305 to 426 aa (Fig. 1A). Moreover, amino acid alignment analysis indicated that Pellino sequence was relatively conserved among insect species, and LsPellino shared a 98.5% aa identity with *Nilaparvata lugens* Pellino (Fig. 1B). Phylogenetic analysis showed that LsPellino was closely related to the homologs of two other planthoppers (*N. lugens* and *Sogatella furcifera*) (Fig. 1C).

### Suppression of LsPellino expression during RSV infection in small brown planthoppers

**Fig 2:**
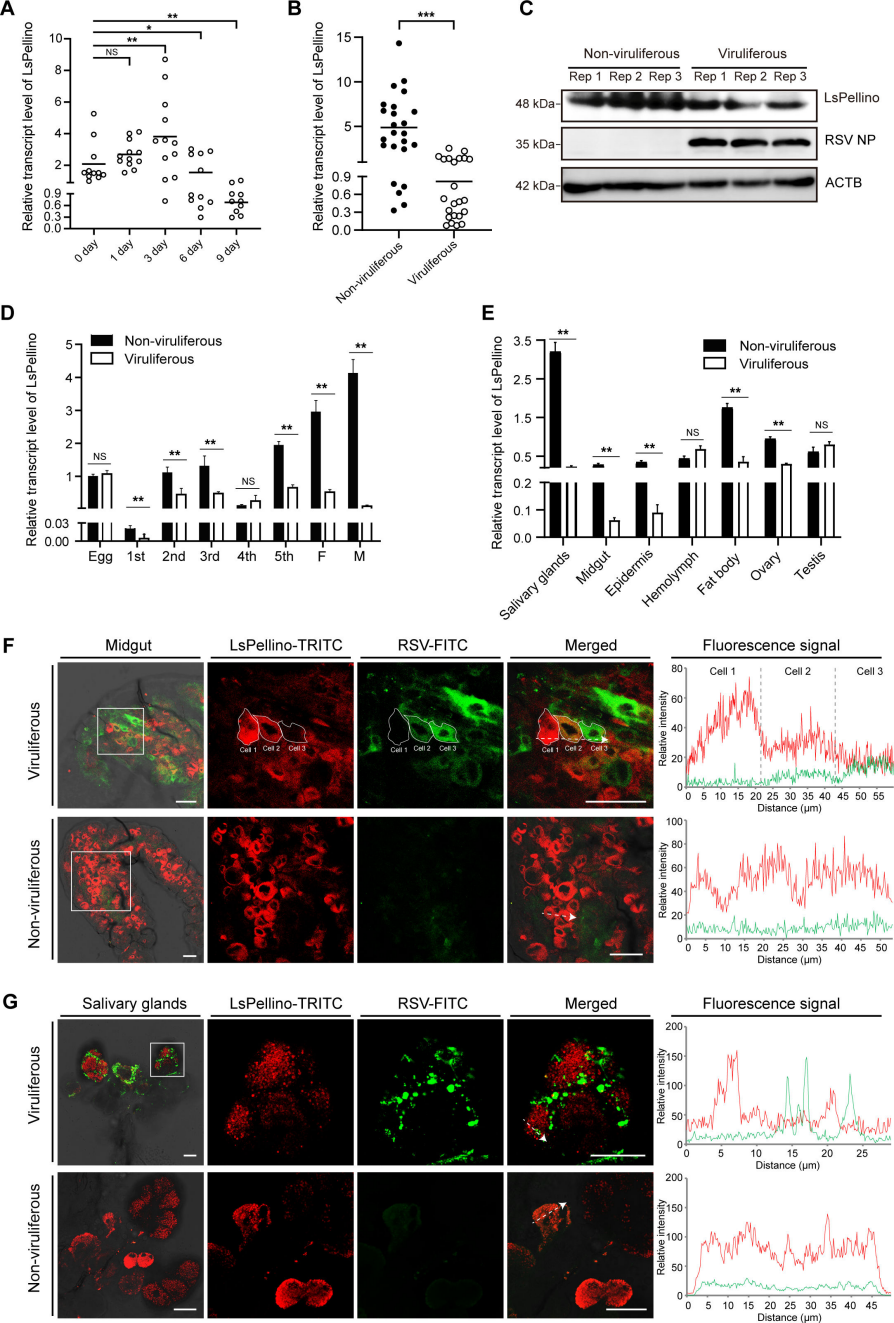
Suppression of LsPellino expression during RSV infection in small brown planthoppers. (**A**) The transcript levels of *LsPellino* in planthoppers at various time points (0, 1, 3, 6, and 9 days) after RSV infection were determined by RT-qPCR. Each dot represents an insect sample. (**B and C**) The expression of LsPellino at both the transcript and protein levels in nonviruliferous and viruliferous planthoppers was analyzed using RT-qPCR (**B**) and western blotting (**C**). Three independent replicates (Rep 1, Rep 2, and Rep 3) from nonviruliferous or viruliferous insects were selected for immunoblotting analysis. The molecular weight is displayed on the left. ACTB was used as a loading control. (**D and E**) RT-qPCR analysis of the expression patterns of *LsPellino* in the samples of nonviruliferous and viruliferous planthoppers at different developmental stages (**D**) and in various tissues (**E**). NS, not significant. *, *P* < 0.05, **, *P* < 0.01 and ***, *P* < 0.001 by the student *t*-test. The error bars represent the standard error of the mean (SEM). Three independent biological replicates were performed for each experiment. (**F and G**) Immunofluorescence labeling of LsPellino (red) and RSV particles (green) in the midgut (**F**) and salivary glands (**G**). LsPellino was detected using a TRITC-conjugated anti-LsPellino antibody. RSV particles were detected using a FITC-conjugated anti-RSV NP antibody. The boxed areas are enlarged and shown on the right. Overlapping fluorescence spectra of LsPellino and RSV in the enlarged areas were analyzed and shown in the right panels. The white-dashed arrows indicate the direction of the line scans and are used to create the fluorescence intensity profiles. Bar, 50 µm.

To investigate whether the *LsPellino* was involved in RSV infection, we first examined the expression of *LsPellino* during RSV acquisition using RT-qPCR. The results indicated that the transcription level of *LsPellino* was upregulated at 1 and 3 days, whereas it was significantly downregulated at 6 and 9 days after feeding on the RSV-infected plants (Fig. 2A). In addition, RT-qPCR and western blotting analyses showed that the expression of *LsPellino* at both the transcriptional and protein levels was decreased in the whole bodies of viruliferous planthoppers compared with nonviruliferous planthoppers (Fig. 2B and C). We then explored the expression pattern of *LsPellino* across different stages and in various organs of the nonviruliferous and viruliferous planthoppers. RT-qPCR revealed that *LsPellino* was expressed in all developmental stages, and the expression level in nonviruliferous planthoppers was higher than that in viruliferous planthoppers (Fig. 2D). Besides, *LsPellino* was ubiquitously expressed in all tissues, and its expression level was obviously reduced in RSV-infected planthoppers compared with non-infected planthoppers (Fig. 2E). Furthermore, immunofluorescence labeling using the anti-LsPellino and anti-RSV NP antibodies showed that LsPellino was not localized with RSV virions, and the fluorescence intensity of LsPellino was significantly reduced in RSV-infected cells compared to uninfected cells in midgut and salivary glands (Fig. 2F and G). These results suggested that the expression of LsPellino is inhibited during RSV infection in small brown planthoppers.

### LsPellino inhibits RSV replication and transmission in small brown planthoppers

**Fig 3:**
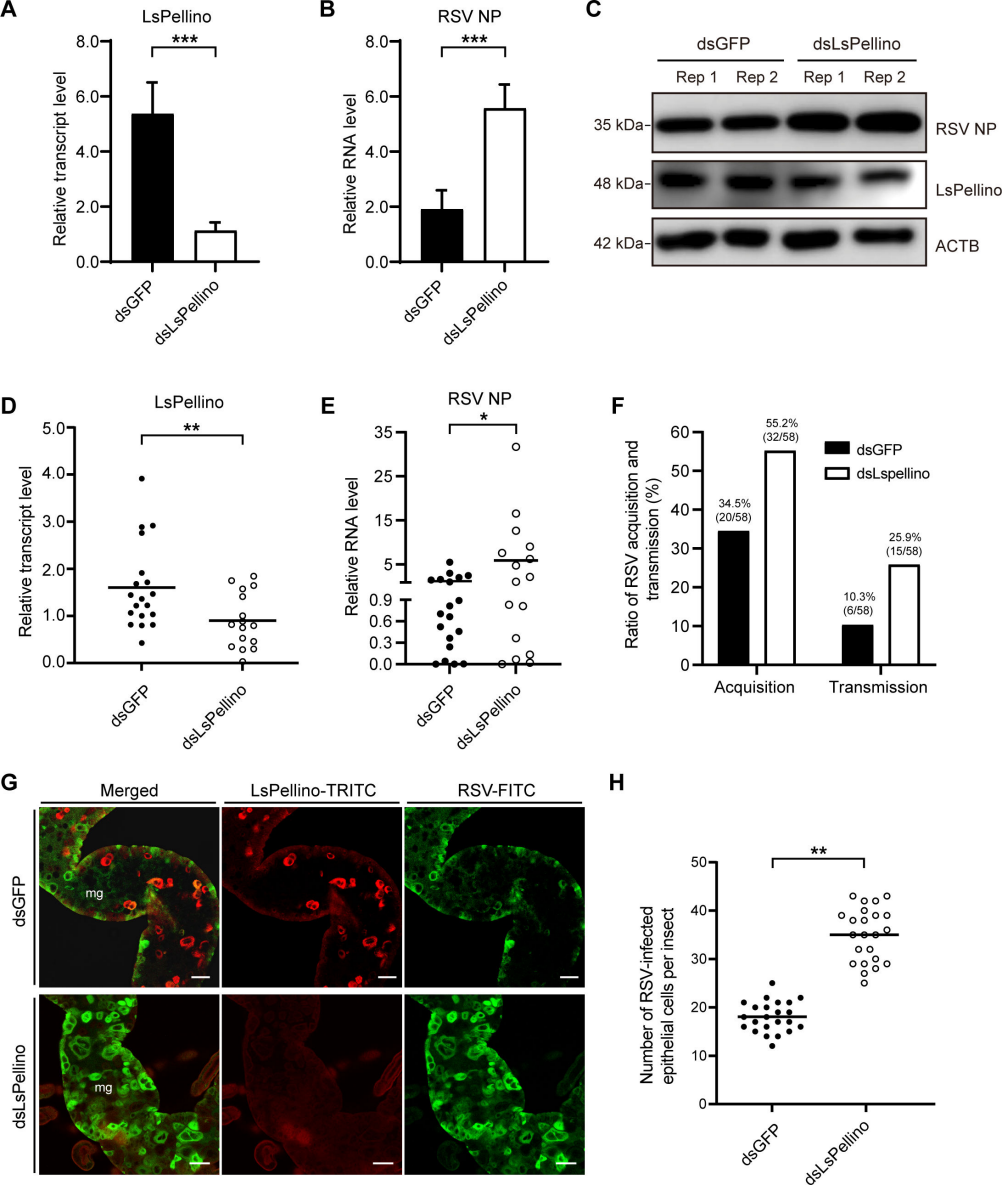
LsPellino inhibits RSV replication and transmission in small brown planthoppers. (**A**) The relative transcript levels of *LsPellino* in ds*GFP*- or ds*LsPellino*-treated viruliferous planthoppers were analyzed using RT-qPCR. (**B and C**) The relative RNA (**B**) and protein levels (**C**) of RSV NP in ds*GFP*- or ds*LsPellino*-treated viruliferous planthoppers were measured by RT-qPCR and western blotting assays. (**D and E**) The relative transcript levels of *LsPellino* (**D**) and RSV NP (**E**) in nonviruliferous planthoppers injected with a mixture of ds*GFP* or ds*LsPellino* and RSV crude extracts were analyzed using RT-qPCR. (**F**) The acquisition and transmission rates of RSV by ds*GFP*- or ds*LsPellino*-treated planthoppers. The ratios are indicated above the bar graph, showing the number of virus infections/total number of insects (plants) in brackets. (**G**) Immunofluorescence staining of LsPellino (red) and RSV particles (green) in the intestines of viruliferous insects treated with ds*GFP*- or ds*LsPellino*. mg, midgut. Bar, 50 µm. (**H**) Statistics on the number of RSV-infected epithelial cells in the intestines of ds*GFP*- or ds*LsPellino*-treated viruliferous insects. Each dot represents an insect sample. *, *P* < 0.05, **, *P* < 0.01, and ***, *P* < 0.001 by the student *t*-test. Three independent biological replicates were performed for each experiment.

After confirming that *LsPellino* was inhibited during RSV infection, we then investigated the role of *LsPellino* in viral infection in planthoppers. Viruliferous planthoppers were first treated with double-stranded *LsPellino* (ds*LsPellino*) or green fluorescent protein (ds*GFP*) for 48 h, resulting in a significant reduction in the relative transcript level of *LsPellino* (Fig. 3A). RT-qPCR and western blotting analyses indicated that the accumulation of RSV nucleocapsid protein (NP) at both RNA and protein levels was increased in the ds*LsPellino*-treated planthoppers compared with the ds*GFP*-treated controls (Fig. 3B and C). When nonviruliferous planthoppers were injected with a mixture of ds*LsPellino* and RSV crude extracts, the expression of *LsPellino* decreased, and the viral RNA level was significantly high compared with the controls (Fig. 3D and E).

Subsequently, we explored the effects of *LsPellino* knockdown on RSV replication and transmission in planthoppers. The results revealed that both the RSV acquisition ratio (55.2%) and transmission ratio (25.9%) in ds*LsPellino*-treated planthoppers were much higher than those in the ds*GFP*-treated controls (Fig. 3F). Immunofluorescence labeling assays showed that the number of RSV-infected midgut epithelial cells was greatly increased in ds*LsPellino*-treated planthoppers compared with the controls (Fig. 3G and H). Together, these results demonstrated that LsPellino inhibits RSV replication and transmission in small brown planthoppers.

### E3 ubiquitin ligase LsSOCS5 interacts with LsPellino and mediates the RSV-induced degradation of LsPellino

**Fig 4:**
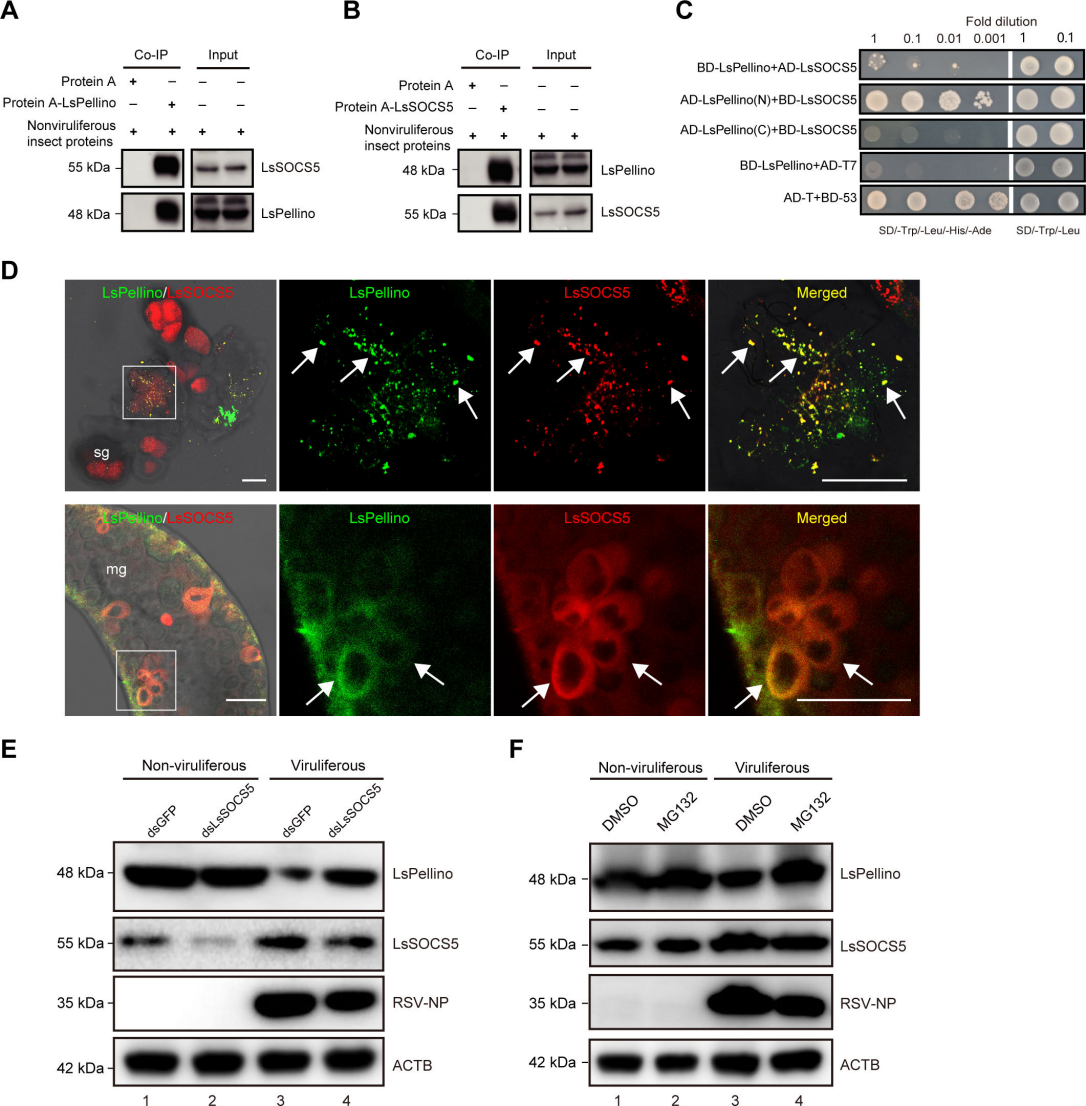
E3 ubiquitin ligase LsSOCS5 interacts with LsPellino and mediates the RSV-induced degradation of LsPellino. (**A and B**) The interaction between LsPellino and LsSOCS5 was confirmed by Co-IP assay. Total protein from nonviruliferous insects was prepared and immunoprecipitated by Protein A-LsPellino (**A**) or Protein A-LsSOCS5 (**B**) combinations. The coimmunoprecipitated proteins were detected with LsSOCS5 and LsPellino antibodies. (**C**) Interaction of LsPellino and LsSOCS5 was confirmed by one-to-one Y2H assay. Yeast cells co-transformed with AD-T and BD-53 were used as a positive control. Yeast co-transformed with BD-LsPellino and AD-T7 served as a negative control. (**D**) Immunofluorescence labeling of LsPellino (green) and LsSOCS5 (red) in the salivary glands and midgut. LsPellino was detected using a FITC-conjugated anti-LsPellino antibody. LsSOCS5 was detected using a TRITC-conjugated anti-LsSOCS5 antibody. The boxed areas are enlarged and shown on the right. The colocalization areas of LsPellino and LsSOCS5 are indicated by arrows. sg, salivary glands; mg, midgut. Bar, 50 µm. (**E**) The protein level of LsPellino in ds*GFP*- or ds*LsSOCS5*-treated nonviruliferous and viruliferous planthoppers was analyzed using western blotting assay. (**F**) Immunoblotting analysis of the expression of LsPellino in nonviruliferous and viruliferous planthoppers treated with DMSO or MG132. ACTB was used as a loading control.

Considering that the expression of LsPellino was reduced in viruliferous planthoppers (Fig. 2), we next investigated which protein was involved in the degradation of LsPellino. Using LsPellino as a bait protein, we screened 72 potential interacting proteins from a cDNA library of *L. striatellus*. Yeast two-hybrid (Y2H) assay showed that an E3 ubiquitin ligase suppressor of cytokine signaling protein 5 in *L. striatellus* (LsSOCS5) was identified as an LsPellino-interacting protein. The interaction between LsPellino and LsSOCS5 was confirmed using an *in vivo* co-immunoprecipitation (Co-IP) assay. The results indicated that the two proteins were mutually precipitated from the nonviruliferous planthoppers using either anti-LsPellino or anti-LsSOCS5 antibodies (Fig. 4A and B). Furthermore, one-to-one Y2H assay revealed that LsSOCS5 specifically interacted with the N-terminal fragment of LsPellino [LsPellino(N), 1–300 aa], but not with the C-terminal fragment of LsPellino [LsPellino(C), 301–442 aa] (Fig. 4C). Immunofluorescence assays further demonstrated that LsPellino and LsSOCS5 were colocalized in the salivary glands and midgut cells of *L. striatellus* (Fig. 4D). We also investigated whether other E3 ubiquitin ligases in *L. striatellus* specifically interacted with LsPellino. Seven E3 ubiquitin ligases were identified by aligning homologous sequences, and one-to-one Y2H assay showed that none of them could interact with LsPellino (Fig. S1). These results suggested that LsSOCS5 may be associated with the degradation of LsPellino.

To better understand the role of LsSOCS5 in LsPellino degradation, viruliferous planthoppers were treated with ds*LsSOCS5* or ds*GFP* for 48 h. Compared with the ds*GFP* controls, the expression level of LsPellino was significantly increased, whereas the viral NP level was markedly decreased in the ds*LsSOCS5*-treated group, as revealed by western blotting (Fig. 4E, lanes 3 vs. 4). Besides, no significant effect on the expression level of LsPellino was detected in the ds*LsSOCS5*-treated nonviruliferous planthopers compared with the controls (Fig. 4E, lanes 1 vs. 2). The 26S proteasome system is one of the primary pathways for protein degradation (37). To investigate whether the proteasome pathway participated in RSV-induced reduction of LsPellino, viruliferous planthopers were treated with the proteasome inhibitor MG132 or dimethyl sulfoxide (DMSO) for 48 h using double-layer parafilm. Western blotting analysis showed that the accumulation level of LsPellino was higher and the accumulation level of RSV NP was lower in the MG132-treated viruliferous planthopers compared with the DMSO-treated controls (Fig. 4F, lanes 3 vs. 4). Meanwhile, MG132 treatment had no significant effect on the accumulation levels of LsPellino in nonviruliferous planthopers, suggesting that the 26S proteasome pathway may be involved in the RSV-induced degradation of LsPellino.

### RSV nonstructural protein NS3 promotes LsSOCS5-mediated degradation of LsPellino

**Fig 5:**
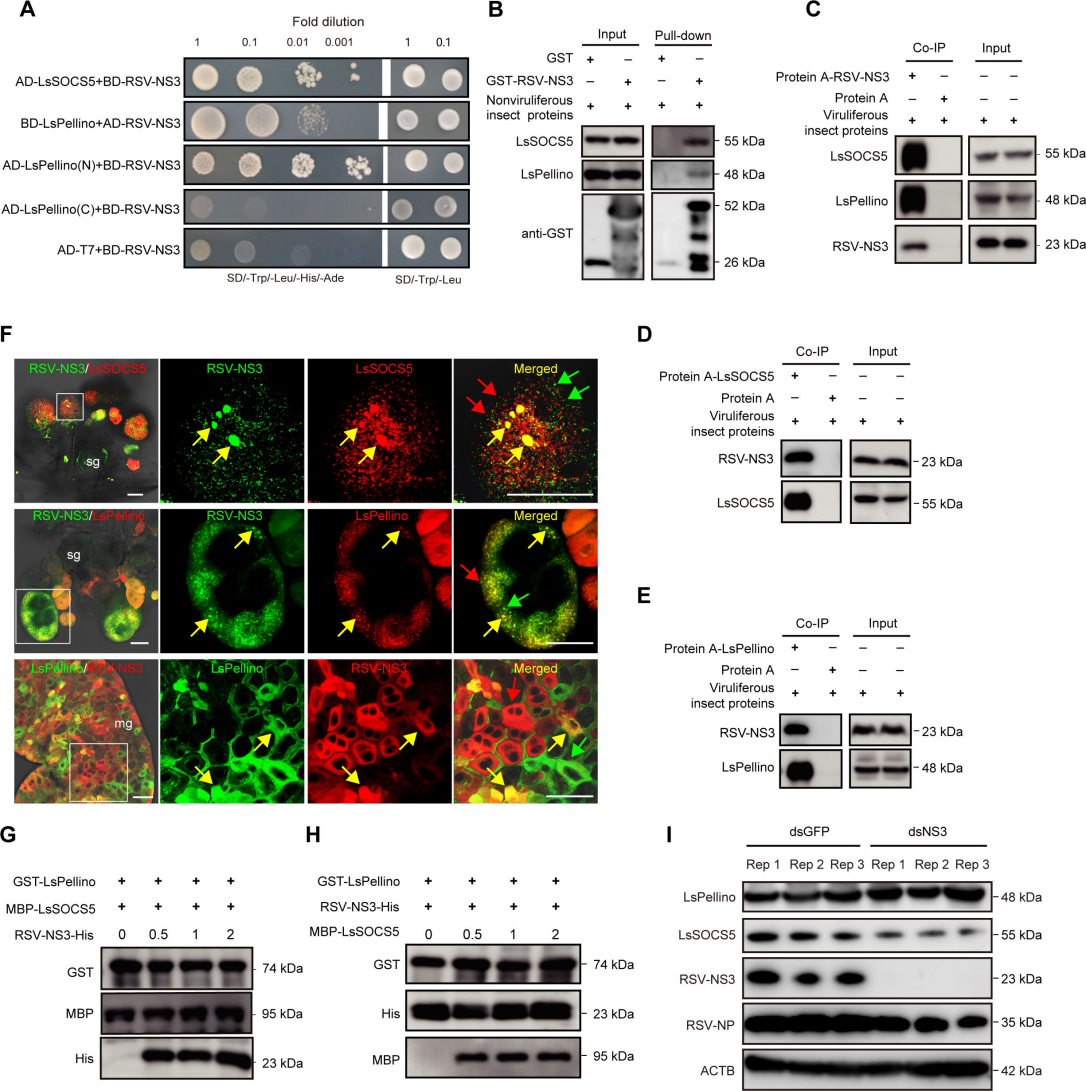
RSV nonstructural protein NS3 promotes LsSOCS5-mediated degradation of LsPellino. (**A**) Interaction between RSV-NS3 and LsSOCS5 or LsPellino in Y2H assay. Yeast co-transformed with BD-RSV-NS3 and AD-T7 was used as a negative control. (**B**) Interaction between RSV-NS3 and LsSOCS5 or LsPellino in GST pull-down assay. The recombinant GST-RSV-NS3 was incubated with GST beads, followed by the addition of total protein from nonviruliferous planthoppers to the beads. The bead-bound proteins were analyzed using western blotting assay. (C–E) Interaction between RSV-NS3 and LsSOCS5 or LsPellino in Co-IP assay. Anti-NS3 (**C**), anti-LsSOCS5 (**D**), and anti-LsPellino (**E**) antibodies were incubated with protein A, followed by total protein from viruliferous planthoppers added to the beads. The coimmunoprecipitated proteins were detected using western blotting assay. (**F**) Immunofluorescence labeling of RSV-NS3, LsSOCS5, and LsPellino in the salivary glands and midgut. LsPellino was detected using a TRITC-conjugated anti-LsPellino antibody. LsSOCS5 was detected using a TRITC-conjugated anti-LsSOCS5 antibody. RSV-NS3 was detected using FITC- or TRITC-conjugated anti-NS3 antibodies. The boxed areas are enlarged and shown on the right. Colocalization areas are indicated by yellow arrows, and non-colocalization areas are indicated by red or green arrows. sg, salivary glands; mg, midgut. Bar, 50 µm. (**G and H**) Competitive binding assay analysis of the interaction among RSV-NS3, LsPellino, and LsSOCS5. GST-LsPellino and MBP-LsSOCS5 were incubated with GST beads, and then RSV-NS3-His was added to the beads. When the amounts of RSV-NS3-His increased, the interaction between LsPellino and LsSOCS5 was not affected (**G**). GST-LsPellino and RSV-NS3-His were incubated with GST beads and then MBP-LsSOCS5 was added to the beads. When the amounts of MBP-LsSOCS5 increased, the interaction between LsPellino and RSV-NS3 was not affected (**H**). (**I**) The protein level of LsPellino in ds*GFP*- or ds*NS3*-treated viruliferous planthoppers was analyzed using western blotting assay. Each group was performed with three independent repetition samples (Rep 1, Rep 2, and Rep 3).

Given that RSV infection could induce the degradation of LsPellino, we next investigated whether viral proteins participated in LsSOCS5-mediated degradation of LsPellino. One-to-one Y2H assay showed that both LsSOCS5 and LsPellino interacted with RSV nonstructural protein NS3 (RSV-NS3). Besides, RSV-NS3 specifically interacted with the LsPellino(N) but not with LsPellino(C) (Fig. 5A). The interactions among RSV-NS3, LsSOCS5, and LsPellino were further confirmed by pull-down and Co-IP assays. LsSOCS5 and LsPellino were pulled down from glutathione S-transferase (GST) beads incubated with recombinant GST-RSV-NS3 protein, but these bands were absent in GST beads incubated with GST protein (Fig. 5B). Additionally, LsSOCS5 and LsPellino were detected in anti-NS3 immunoprecipitates but not in controls (Fig. 5C). Similarly, a significantly higher amount of RSV-NS3 was precipitated from the viruliferous planthoppers using either anti-LsSOCS5 or anti-LsPellino antibodies (Fig. 5D and E). Immunofluorescence microscopy of RSV-infected salivary glands or midguts revealed that RSV-NS3 partially colocalized with LsSOCS5 or LsPellino (Fig. 5F, yellow arrows). Many fluorescent signals without colocalization were also observed (Fig. 5F, red and green arrows). Subsequently, competitive binding assays were performed to determine whether RSV-NS3 could affect the interaction between LsSOCS5 and LsPellino. The results indicated that RSV-NS3 did not compete with LsSOCS5 or LsPellino. Instead, RSV-NS3 could stabilize the interaction between LsSOCS5 and LsPellino (Fig. 5G and H), indicating that RSV-NS3 may play a positive regulatory role in accelerating the degradation of LsPellino. To test this hypothesis, viruliferous planthopers were treated with ds*NS3* and ds*GFP* for 48 h. Western blotting analysis revealed that the expression level of LsPellino was significantly increased in the ds*NS3*-treated planthopper compared with the ds*GFP*-treated controls. Taken together, these results suggested that NS3, LsSOCS5, and LsPellino may form a complex during RSV infection, with NS3 promoting LsSOCS5-mediated degradation of LsPellino.

### The degradation of LsPellino attenuates Toll pathway-mediated antiviral immune response

**Fig 6:**
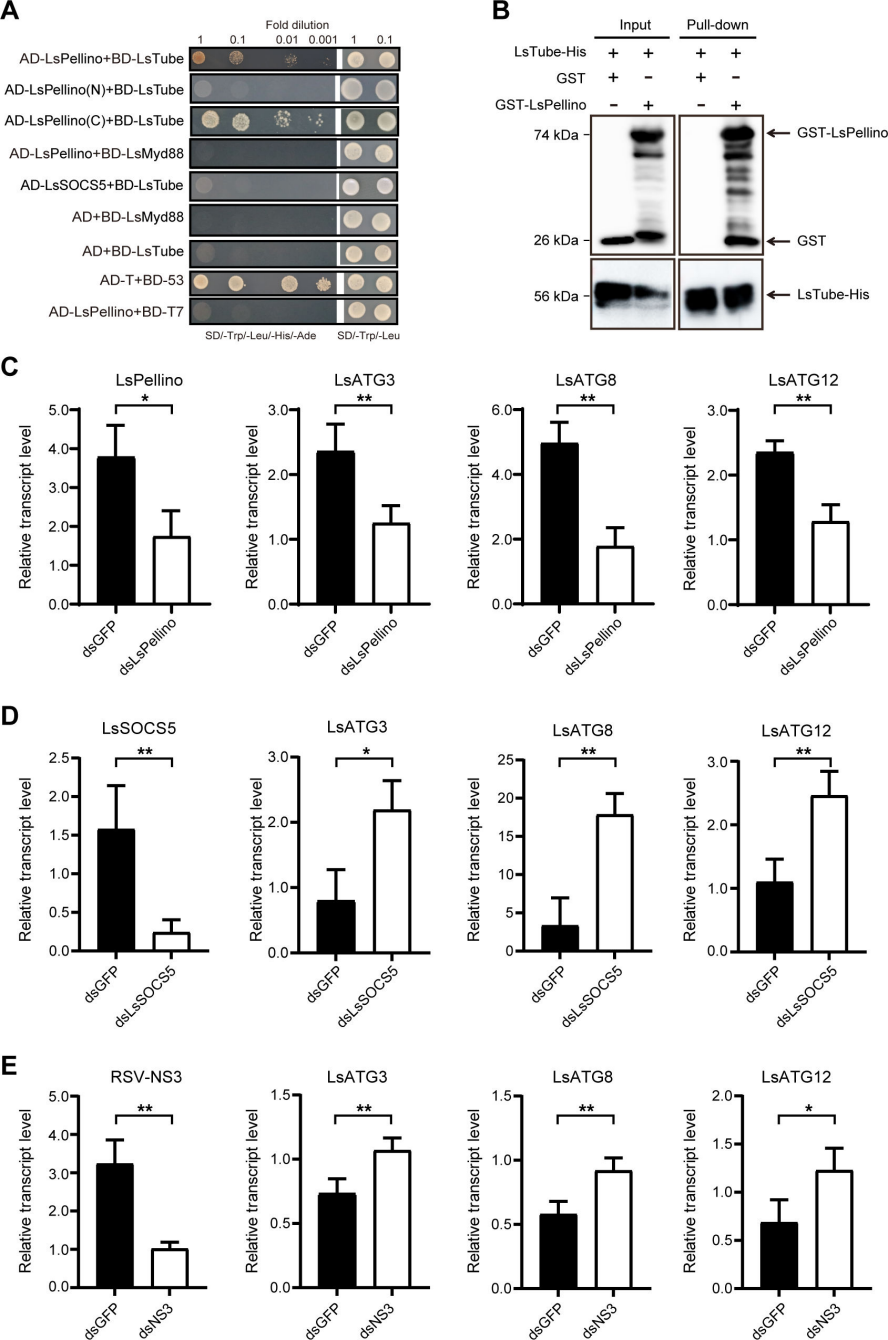
The degradation of LsPellino attenuates Toll pathway-mediated antiviral immune response. (**A**) Interaction between LsPellino and LsTube in Y2H assay. The positive interactions were analyzed in 10-fold serial dilutions in synthetic dextrose (SD) quadruple dropout medium (SD/-Trp/-Leu/-His/-Ade) plates. Yeast cells co-transformed with AD-T and BD-53 were used as a positive control. Yeast co-transformed with AD-LsPellino and BD-T7 served as a negative control. (**B**) Interaction between LsPellino and LsTube in pull-down assay. Recombinant LsTube-His was incubated with Ni-NTA agarose beads, followed by the addition of GST-LsPellino to the beads. The bead-bound proteins were analyzed using western blotting assay. (C–E) The transcript levels of immune-related genes (LsATG3, LsATG8, and LsATG12) in nonviruliferous insects treated with ds*LsPellino* (**C**), ds*LsSOCS5* (**D**), or ds*NS3* (**E**) after treatment with RSV crude extracts were analyzed using RT-qPCR. *, *P* < 0.05 and **, *P* < 0.01 by the student *t*-test. Three independent biological replicates were performed for each experiment.

The Pellino family has been reported to play a crucial regulatory role in Toll/TLR innate immune signaling in both insects and mammals (9, 38). To determine whether LsPellino protein participates in Toll immune signaling in *L. striatellus*, we used one-to-one Y2H and pull-down assays to verify the LsPellino-interacting proteins in the Toll immune pathway. The results showed that LsTube, a core component of Toll immune pathway, could specifically interact with LsPellino (C) and failed to interact with LsPellino (N) (Fig. 6A and B), suggesting that LsPellino most likely had a regulatory effect on Toll immune pathway. Our previous studies have demonstrated that RSV infection activates the Toll signaling, which regulates several downstream antiviral immune responses (including autophagy) to induce antiviral response (35, 36). To test whether LsPellino affects Toll pathway-mediated antiviral immunity, nonviruliferous planthoppers were treated with ds*LsPellino* for 48 h and then with RSV crude extracts for an additional 4 days. Compared with the control, the transcript levels of downstream immune-related genes (LsATG3, LsATG8, and LsATG12) were significantly lower in the ds*LsPellino*-treated groups (Fig. 6C). However, the expression levels of these immune-related genes were not markedly changed in nonviruliferous planthoppers treated with ds*GFP* or ds*LsPellino* (Fig. S2). Given that LsSOCS5 mediates the RSV-induced degradation of LsPellino, we further examined whether LsSOCS5 has an inhibitory effect on antiviral response. RT-qPCR analysis revealed that the expression of these immune genes was significantly increased after ds*LsSOCS5* treatment compared with the ds*GFP* control (Fig. 6D). Consistently, when nonviruliferous planthoppers were treated with ds*NS3* followed by treating with RSV crude extracts, the expression of these immune genes was also notably upregulated compared with the control (Fig. 6E). These results demonstrate that LsSOCS5-mediated degradation of LsPellino attenuates Toll pathway-mediated antiviral immune response.

### LsPellino participates in other rice virus infection in small brown planthoppers

To further investigate whether LsPellino affects the infection of other viruses, rice black-streaked dwarf virus (RBSDV), which is also persistently transmitted by the small brown planthopper, was evaluated in this study. RT-qPCR and western blotting analyses showed that the expression of *LsPellino* at both the transcriptional and protein levels was significantly lower in the RBSDV-infected planthoppers compared with non-infected planthoppers (Fig. S3A and B). In addition, nonviruliferous planthoppers were treated with ds*LsPellino* for 48 h and then with RBSDV crude extracts for another 6 days. The accumulation level of RBSDV capsid protein (RB-P10) was markedly increased compared with the ds*GFP*-treated controls (Fig. S3C). We then investigated whether any other viral proteins could interact with LsPellino or LsSOCS5. Y2H and pull-down assays showed that a minor core capsid protein of RBSDV (RB-P8) could bind to LsPellino and LsSOCS5 (Fig. S3D through F). Thus, these data indicated that LsPellino participates in the infection of RBSDV in small brown planthoppers.

## DISCUSSION

**Fig 7:**
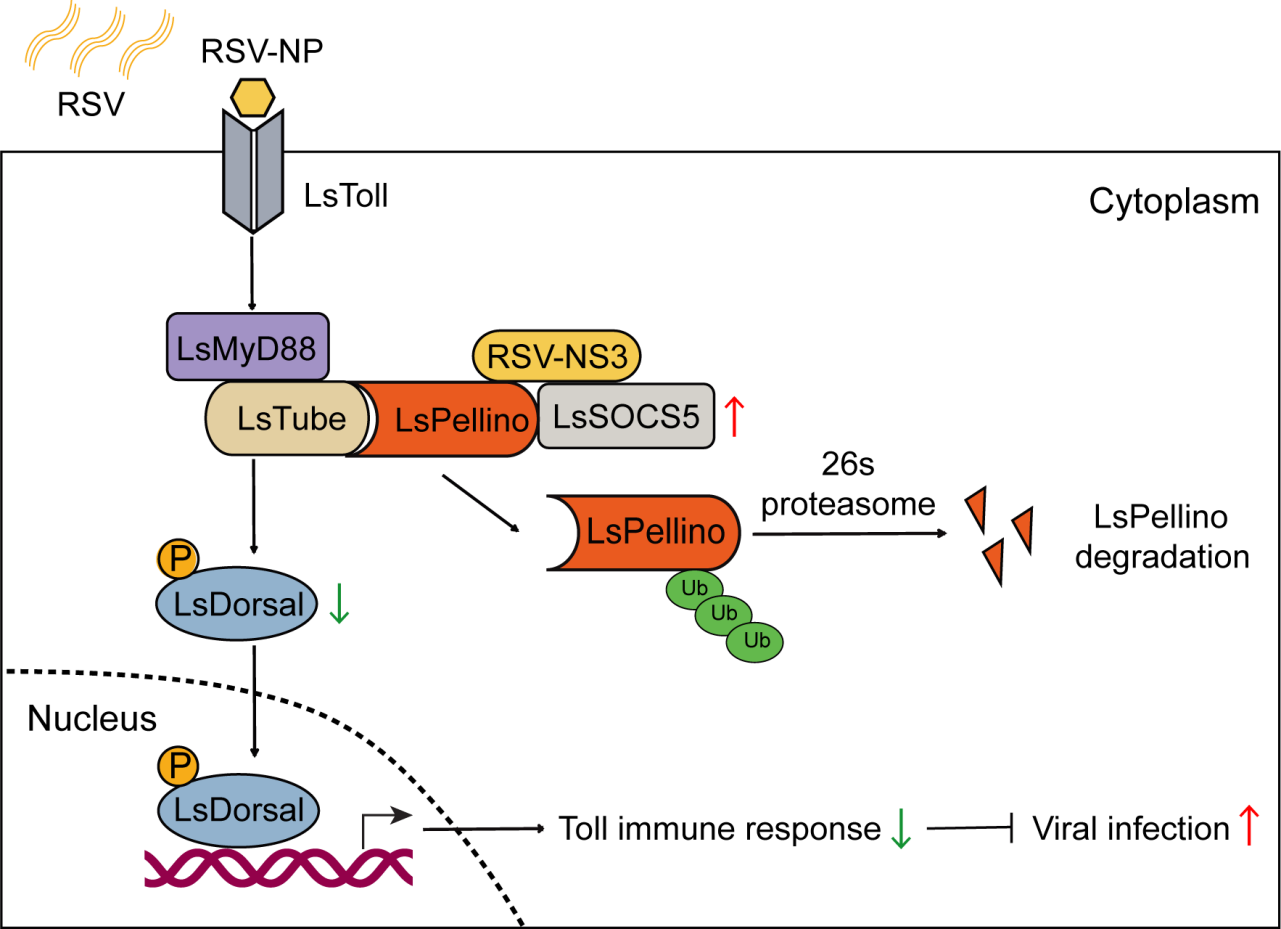
The schematic diagram indicating viral protein attenuating the Toll antiviral pathway via promoting degradation of LsPellino. RSV infection activates the Toll pathway by the interaction of RSV-NP and Toll receptor (LsToll) (35, 36). Subsequently, LsPellino participates in the Toll antiviral pathway by interacting with LsTube and induces the downstream immune response (such as autophagy). Meanwhile, RSV-NS3 hijacks LsSOCS5 to mediate the degradation of LsPellino through the 26S proteasome pathway, finally attenuating the Toll antiviral pathway to facilitate RSV infection in insect vectors.

During the long-term evolutionary process, plant viruses have developed various counter-defense strategies to suppress the innate immune system of insect vectors and maintain persistent infection and transmission (39–43). Our previous results indicated that RSV-NP binds to the cell surface receptor LsToll and activates the Toll signaling pathway, which subsequently activates the downstream transcription factor LsDorsal and regulates the antiviral immune response. In contrast, RSV utilizes its NS4 protein to inhibit LsDorsal phosphorylation by competitively binding to LsMSK2 kinase, thereby counteracting Toll antiviral immunity (35, 36). In this study, we identified a novel anti-defense strategy to antagonize Toll antiviral defense (Fig. 7). LsPellino participates in the Toll immune pathway by interacting with LsTube and activates a downstream immune response to inhibit viral infection. Meanwhile, RSV-NS3 hijacks LsSOCS5 to promote the degradation of LsPellino through the 26S proteasome pathway, finally attenuating the Toll antiviral pathway to facilitate RSV infection in insect vectors (Fig. 7).

The Pellino family is regarded as scaffold proteins in the signal transduction process due to their interactions with multiple intermediates (44–46). In *Drosophila*, Pellino enhances innate immunity by interacting with Pelle and acts as a negative regulator of the Toll signaling pathway by targeting MyD88 for ubiquitination and degradation. Our study reveals that LsPellino cannot bind to LsMyD88 but interacts with LsTube (Fig. 6A). Although the degradation of LsPellino occurs via the proteasome pathway, we cannot rule out the possibility that other degradation pathways are involved in LsPellino degradation, such as the autophagy pathway. Moreover, our results showed that the downstream immune-related genes are downregulated after knockdown of LsPellino expression. Whether the interaction between LsPellino and LsTube affects the degradation of LsCactus and the phosphorylation of LsDorsal still needs to be further elucidated. Besides, the Pellino family can be phosphorylated by multiple intermediates after interaction, thereby activating Pellino proteins and regulating downstream signaling (11, 38, 47). For example, Pellino recognizes phosphorylated IRAKs (IL-1R-associated kinases) through its N-terminal FHA domain, and Pellino is phosphorylated by IRAKs, which enhances the E3 ubiquitin ligase activity of Pellino (14, 48, 49). A mutation in the Pellino C-terminal RING-like domain results in the loss of E3 ligase activity (48). Furthermore, kinase-active IRAKs promote polyubiquitination and degradation of the Pellino family, whereas loss of kinase-active IRAKs increases the expression level of Pellino (50–52). Our results indicated that the RING domain of LsPellino, but not the N-terminal FHA domain, can bind to LsTube. Whether LsTube can catalyze the phosphorylation of LsPellino and subsequently lead to LsTube-dependent polyubiquitination and degradation of LsPellino remains to be further explored.

Recent studies have reported that many viruses affect the function of Pellino to evade host antiviral immunity in both insects and mammals. In *Drosophila*, the poxvirus homolog of the Pellino binds to IRAKs and suppresses Toll- and TLR-mediated activation of downstream transcription factors (53). In the mammalian system, the microRNA-155 is upregulated and targets mRNA to inhibit the expression of Pellino during the Japanese encephalitis virus infection (54). In our study, we showed that the nonstructural protein NS3 of RSV directly interacts with the LsPellino N-terminal FHA domain and promotes LsSOCS5-mediated degradation of LsPellino (Fig. 5). It will be interesting in future studies to determine the specific amino acid sequences or domains of RSV-NS3 that bind to LsSOCS5 and LsPellino. Besides, we also revealed that the expression level of LsPellino is reduced following RBSDV infection, and the RBSDV P8 protein also binds to both LsPellino and LsSOCS5 (Fig. 7). Targeting Pellino to attenuate the Toll antiviral pathway may represent a universal mechanism for ensuring the persistent transmission of other viruses by their insect vectors.

Our previous studies have demonstrated that RSV and RBSDV infection can activate the JAK-STAT pathway, promote the accumulation of LsSOCS5 regulated by the transcription factor STAT5B, and facilitate persistent viral infection in insect vectors (34). Our results showed that RSV-NS3 hijacks LsSOCS5 to promote the degradation of LsPellino, thereby attenuating the Toll antiviral pathway. These two pathways appear to be antagonistic to one another. An intriguing study will focus on how viruses manipulate these two pathways to achieve homeostasis during persistent viral infection. For different methods of virus inoculation (e.g., feeding vs. injection) may result in differences in virus infectivity and vector fitness. For example, injection can increase not only virus infectivity but also insect mortality, whereas feeding reduces virus infectivity but has no significant effect on vector fitness. Moreover, immunofluorescence microscopy of RSV-infected salivary glands or midguts revealed that RSV does not infect all host cells but accumulates only in localized cells (Fig. 2). Other immune pathways, such as siRNAi, PPO, and JNK, have also been reported to participate in viral infection in planthoppers (31–33). Whether interactions exist among these immune regulatory networks to limit excessive viral accumulation warrants further investigation.

In summary, we revealed that LsPellino is involved in the Toll immune pathway by interacting with LsTube and activates a downstream immune response to inhibit viral infection. Meanwhile, RSV-NS3 hijacks LsSOCS5 to promote the degradation of LsPellino via the 26S proteasome pathway, ultimately attenuating the Toll antiviral pathway to facilitate RSV infection in insect vectors. Our findings uncover a novel counter-defense strategy that may expand the understanding of the arms race between viruses and insect vectors during coevolution.

## MATERIALS AND METHODS

### Insects and rice viruses

The population of small brown planthopper was reared on rice seedlings of rice at 26 ± 1°C with a photoperiod of 14 h light and 10 h dark. The RSV-infected and RBSDV-infected rice plants originated from Jiangsu Province, China. The RSV-infected or RBSDV-infected planthoppers were obtained by feeding on virus-infected plants or injecting virus crude extracts. To ensure that the proportion of RSV-carrying (viruliferous) planthoppers exceeds 90%, the viruliferous population was regularly screened (every 3 months) using RNA extraction from individual insects and PCR detection according to the previous description (55).

### Total RNA extraction and cDNA synthesis

Total RNA was extracted from the whole bodies of insects or from seven tissues (salivary gland, midgut, epidermis, hemolymph, fat body, ovary, and testis) using TRIzol reagent (Invitrogen, Carlsbad, CA, USA). The concentration and quality of total RNA were assessed using a NanoDrop 2000 spectrophotometer (Thermo Fisher Scientific, Waltham, MA, USA). One microgram of RNA from whole bodies or from each tissue was reverse transcribed to cDNA using the HiScript II Q RT SuperMix for qPCR (+gDNA wiper) (Vazyme, China).

### Gene cloning and sequence analysis

*LsPellino* and other genes were amplified from the cDNA template based on the genomic information for the *L. striatellus* (56). Two sequences of RSV (*NP* and *NS3*) and two sequences of RBSDV (*P8* and *P10*) were amplified from the cDNA of insects infected with these two viruses. The functional domain of *LsPellino* was predicted using the Conserved Domain Database server (57). The amino acid sequence alignment of Pellino from *L. striatellus* and other insect species was conducted with the BioEdit 7.2 program. The phylogenetic tree was constructed using a Machine Learning Algorithm via the RAxML-NG program with 1,000 bootstrap replicates (58).

### Real-time quantitative PCR analysis

Real-time quantitative PCR (RT-qPCR) was performed to evaluate the relative RNA levels of viral or insect genes. A 10 µL reaction mixture, consisting of 5 µL of Hieff qPCR SYBR Green Master Mix (11202ES08, Yeasen, China), 4 µL of 20-fold diluted cDNA template, and 0.5 µL of each primer (10 µM), was conducted on a LightCycler Real-Time PCR System (Roche Swiss). The primers utilized for the RT-qPCR are listed in Table S1. The transcript level of the *Actin* gene served as an internal reference to quantify the expression of each gene in *L. striatellus*. Each experiment was performed with three biological replicates, with each biological replicate containing two technical replicates.

### Injection of virus crude extracts

The virus crude extracts were prepared as described previously (34). Briefly, a total of 10 RSV-infected insect samples were collected and homogenized in 100 µL of 10 mM phosphate-buffered saline (PBS, pH 7.4) in a 1.5 mL tube. After centrifugation at 4°C and 8,000 rpm for 5 min, the supernatant was transferred to another EP tube and centrifuged three additional times. The supernatant sample from the final centrifugation was regarded as the virus crude extracts. A volume of 25 nl of virus crude extracts was injected into each nonviruliferous third-instar planthoppers using a TransferMan 4 r micromanipulator (Eppendorf, Hamburg, Germany). Each replicate included 50 nonviruliferous insects, and three replicates were conducted for each experiment.

### Double-stranded RNA synthesis and delivery

The double-stranded RNAs (dsRNAs) targeting *LsPellino*, *LsSOCS5*, *NS3,* and green fluorescent protein gene (*GFP*) were synthesized using the T7 High Yield RNA Synthesis Kit (10623ES60, Yeasen, China) following the manufacturer’s protocol. The purity and integrity of these dsRNAs were evaluated using agarose gel electrophoresis. A volume of 20 nl of dsRNA was injected into the insect’s ventral thorax through a glass needle with a TransferMan 4 r micromanipulator. Viral loads at the RNA level and transcription levels of insect genes were quantified using RT-qPCR. The protein levels of RSV NP, NS3, RBSDV P10, LsPellino, and LsSOCS5 were determined by western blotting with specific antibodies.

### Western blotting assay

Protein samples were extracted from 20 planthoppers in 200 µL of 10 mM PBS (pH 7.4) and boiled with a loading buffer at 95°C for 10 min. After being separated in 10% (wt/vol) SDS-PAGE gels, the samples were transferred onto polyvinylidene fluoride (PVDF) membranes. The blots were incubated with specific primary antibodies at a dilution of 1:5,000 (vol/vol), followed by the application of horseradish peroxidase (HRP)-conjugated secondary antibodies at a dilution of 1:10,000 (vol/vol). The membranes were treated with SuperSignal West Pico PLUS chemiluminescent substrate (34577, Thermo Scientific, USA) and visualized using a CCD camera system Amersham Imager 680 (GE, Sweden).

Specific primary antibodies against LsPellino, RSV NP, NS3, or RBSDV P10 were produced in the laboratory. The anti-SOCS polyclonal antibody (HA500307) and anti-beta Actin monoclonal antibody (EM21002) were acquired from Huabio (Hangzhou, China) and used to quantify LsSOCS5 and ACTB (beta-actin), respectively. MBP-tag monoclonal antibody (MA5-27544), GST-tag monoclonal antibody (MA4-004), and His-tag monoclonal antibody (MA1-21315) were obtained from Invitrogen (Carlsbad, CA, USA) and used in competitive binding assay.

### Enzyme-linked immunosorbent assay

The rates of RSV acquisition and transmission were determined by enzyme-linked immunosorbent assay. The collected planthoppers or rice plants were ground individually in PBS buffer. Then, the samples were centrifuged at 8,000 rpm for 5 min, and the supernatants from each sample were blotted on the nitrocellulose membranes. After drying at room temperature for 10 min, the membranes were incubated with an RSV NP antibody at a dilution of 1:5,000 (vol/vol), followed by the application of HRP-conjugated goat anti-rabbit antibody at a dilution of 1:10,000 (vol/vol). The membranes were treated with SuperSignal West Pico PLUS chemiluminescent substrate and visualized using a CCD camera system Amersham Imager 680 (GE, Sweden).

### Competitive binding assay

The full-length ORF of LsPellino was cloned into the pGEX-6P-1 vector and recombinantly expressed as a GST fusion protein (GST-LsPellino). LsSOCS5 was cloned into the pMAL-c5x vector to express the recombinant protein with the MBP tag (MBP-LsSOCS5). The RSV NS3 gene was cloned into the pET-28a vector for expression of a His-tagged recombinant protein (RSV-NS3-His). Recombinant proteins GST-LsPellino, MBP-LsSOCS5, or RSV-NS3-His were expressed in the *Escherichia coli* strain BL21 (DE3) and purified *in vitro*, respectively. GST-LsPellino was first incubated with GST-Sefinose Resin beads (Sangon, Shanghai, China) for 2 h, and then, MBP-LsSOCS5 was added and incubated for 4 h at 4°C. Subsequently, varying amounts of RSV-NS3-His were added to the beads and incubated for another 4 h at 4°C. In a separate experiment, GST-LsPellino was first incubated with GST-Sefinose Resin beads for 2 h, followed by the sequential addition of RSV-NS3-His and varying amounts of MBP-LsSOCS5 and incubated for 4 h at 4°C. The bead-bound proteins were boiled with protein loading buffer and detected by western blotting using GST-tag, MBP-tag, and His-tag antibodies.

### Pull-down assay

The recombinant His-tagged LsTube protein (LsTube-His) was incubated with Ni-NTA agarose beads (Qiagen, Germany) at 4°C for 2 h. The beads were centrifuged for 5 min at 2,500 rpm and washed three times with PBS buffer. The recombinant protein GST or GST-LsPellino was then added to the beads and inoculated at 4°C for 4 h. In another experiment, the recombinant GST-LsSOCS5 or GST-LsPellino proteins were bound to glutathione S-transferase (GST) beads (Sangon, Shanghai, China) at 4°C for 2 h, followed by MBP and MBP-RB-P8 proteins being added to the beads and inoculated at 4°C for 4 h. After centrifugation and six washes with PBS buffer, the mixtures were eluted by boiling in loading buffer for 10 min and analyzed by western blotting using anti-GST and anti-His antibodies.

The recombinant GST-RSV-NS3 or GST proteins were bound to GST beads at 4°C for 2 h. After being centrifuged and washed three times with PBS buffer, 500 µL of total protein from nonviruliferous planthoppers was added to the beads and inoculated at 4°C for 4 h. The bead-bound proteins were eluted with protein loading buffer and analyzed by western blotting using anti-GST, anti-LsPellino, and anti-LsSOCS5 antibodies.

### Co-immunoprecipitation assay

Five micrograms of anti-LsPellino, anti-LsSOCS5, or anti-NS3 polyclonal antibody were first incubated with 20 µL protein A agarose (Roche, Basel, Switzerland) for 2 h at 4°C. The mixture was centrifuged for 5 min at 2,500 rpm, and the supernatants were discarded. After washing six times with PBS buffer, 500 µL of total protein from nonviruliferous or viruliferous planthoppers in PBS buffer was then added and inoculated with agarose beads for 4 h at 4°C. The antibody-protein complex was eluted from the beads with a protein loading buffer. The eluted samples were separated by SDS-PAGE gels and detected using anti-LsPellino, anti-LsSOCS5, or anti-NS3 antibodies.

### Immunofluorescence labeling of salivary glands and midguts

Salivary glands and midguts were dissected from nonviruliferous or viruliferous insects in PBS buffer (pH 7.4) and fixed in 4% paraformaldehyde for 1 h. The samples were washed three times with PBS buffer and permeabilized in 2% Triton X-100 solution for 30 min. Subsequently, the samples were incubated with tetramethylrhodamine (TRITC)-conjugated anti-LsPellino antibody and fluorescein (FITC)-conjugated anti-RSV NP antibody at a dilution of 1:100 (vol/vol). In another group, the samples were incubated with FITC-conjugated anti-NS3 antibody and TRITC-conjugated anti-LsPellino antibody or TRITC-conjugated anti-LsSOCS5 antibody. After washing three times with PBS buffer, the tissues were examined under a Leica TCS SP8 confocal microscope (Leica Microsystems, Solms, Germany).

### Yeast two-hybrid assay

For the yeast two-hybrid (Y2H) screen, the ORF of *LsPellino* was amplified and fused with the Gal4 DNA binding domain (BD) of the bait plasmid pGBKT7. A cDNA library of *L. striatellus* was constructed and fused with the Gal4 DNA activating domain (AD) of the prey plasmid pGADT7. These two plasmids were co-transformed into the yeast strain AH109. The putative positive clones were selected on synthetic dextrose (SD) quadruple dropout medium (SD/-Trp/-Leu/-His/-Ade) plates and isolated for Sanger sequencing.

For one-to-one Y2H, the ORFs of *LsPellino*, *LsSOCS5*, *LsTube*, *RSV-NS3,* and *RB-P8* were individually cloned into either pGBKT7 or pGADT7. These two plasmids were co-transformed into AH109 and grown on a double dropout medium (SD/-Trp/-Leu). The yeast cells were then transferred to an SD/-Trp/-Leu/-His/-Ade plate and grown for 4 days to observe protein-protein interactions.

## Data Availability

The data that support the findings of this study are available on request from the corresponding author.

## SUPPLEMENTAL MATERIAL

Fig. S1 jvi.00021-25-s0001.tif Y2H assay analysis of LsPellino and other E3 ubiquitin ligases in *L. striatellus*.

Fig. S2 jvi.00021-25-s0002.tif The transcript levels of immune-related genes (LsATG3, LsATG8, LsATG12) in nonviruliferous treated with dsLsPellino were analyzed using RT-qPCR.

Fig. S3 jvi.00021-25-s0003.tif LsPellino participates in other rice virus infection in small brown planthoppers.

Supplemental legends jvi.00021-25-s0004.docx Legends for Fig. S1 to S3.

Table S1 jvi.00021-25-s0005.docx Primers used in this study.

ASM does not own the copyrights to Supplemental Material that may be linked to, or accessed through, an article. The authors have granted ASM a non-exclusive, world-wide license to publish the Supplemental Material files. Please contact the corresponding author directly for reuse.
