# Supplementary figures and images for: A plant virus attenuates the Toll immune pathway by degradation of Pellino to facilitate viral infection in insect vectors
Source: J Virol. 2025 Mar 31;99(5):e00021-25. doi: 10.1128/jvi.00021-25 (PMC12090757; doi:10.1128/jvi.00021-25)

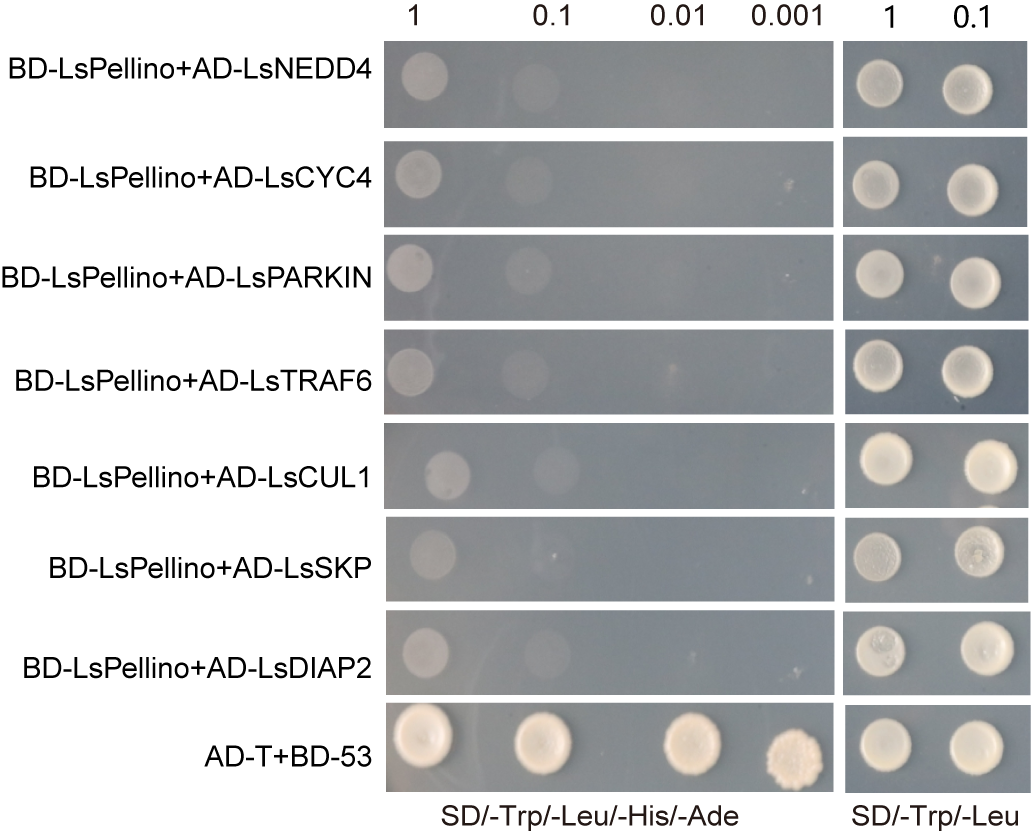

Supplement: Fig. S1 — Y2H assay analysis of LsPellino and other E3 ubiquitin ligases in L. striatellus. [file jvi.00021-25-s0001.tif]

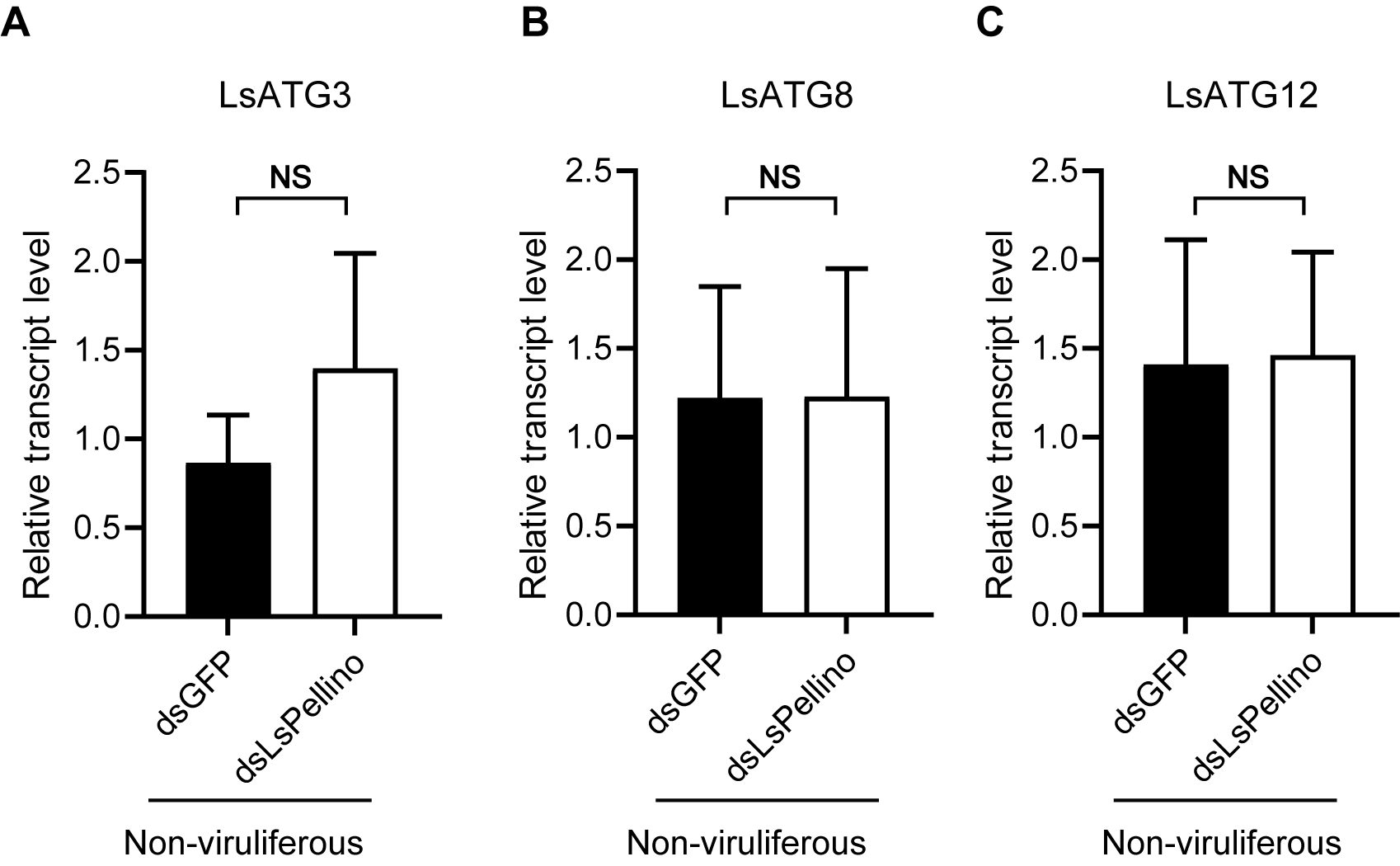

Supplement: Fig. S2 — The transcript levels of immune-related genes (LsATG3, LsATG8, LsATG12) in nonviruliferous treated with dsLsPellino were analyzed using RT-qPCR. [file jvi.00021-25-s0002.tif]

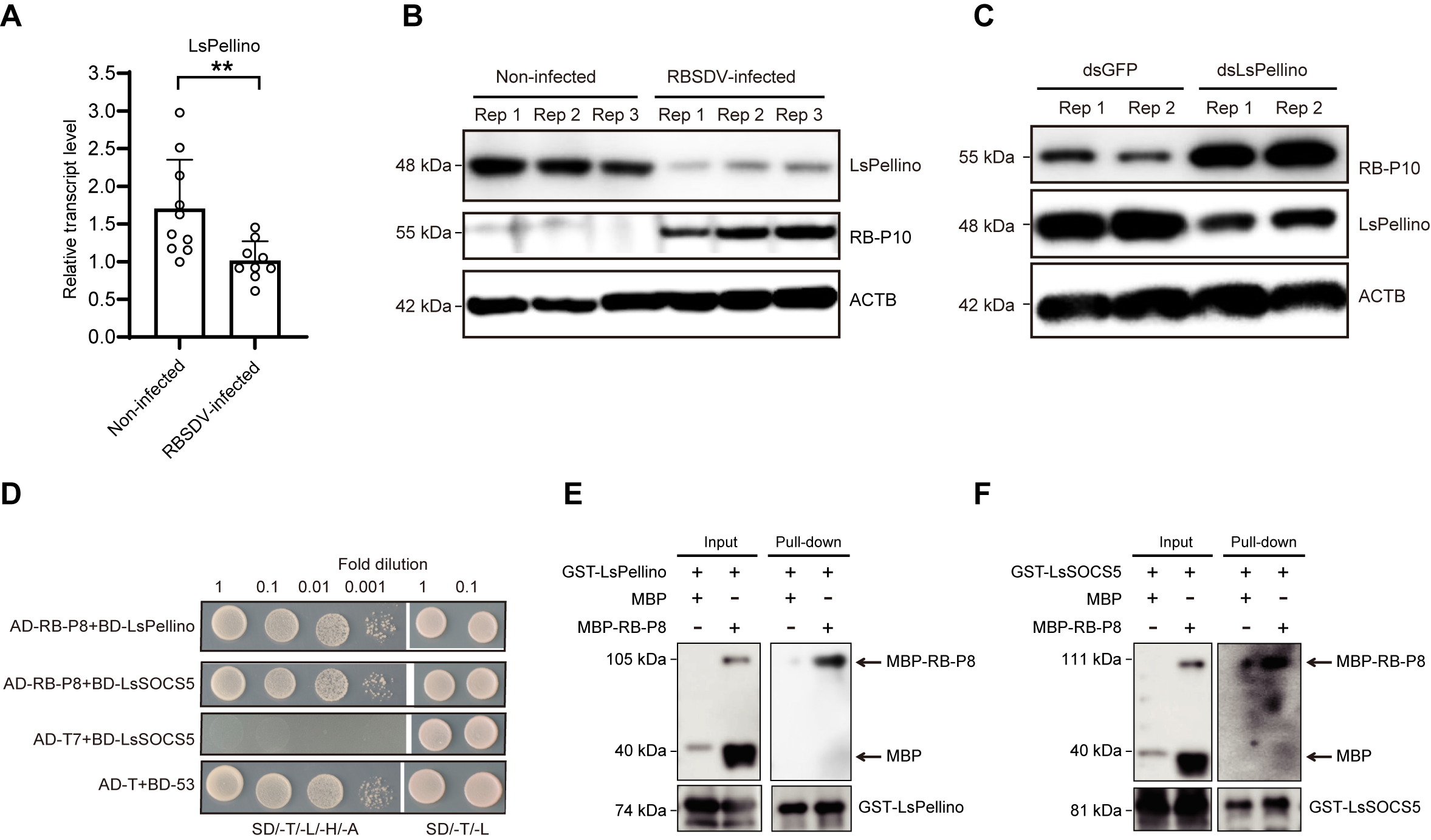

Supplement: Fig. S3 — LsPellino participates in other rice virus infection in small brown planthoppers. [file jvi.00021-25-s0003.tif]
