# Supplementary material for: A plant virus attenuates the Toll immune pathway by degradation of Pellino to facilitate viral infection in insect vectors
Source: J Virol. 2025 Mar 31;99(5):e00021-25. doi: 10.1128/jvi.00021-25 (PMC12090757; doi:10.1128/jvi.00021-25)
Supplement: Supplemental legends — Legends for Fig. S1 to S3. [file jvi.00021-25-s0004.docx]

**Supplementary Material**

**Fig. S1.** Y2H assay analysis of LsPellino and other E3 ubiquitin ligases in *L. striatellus*. Yeast cells co-transformed by AD-T and BD-53 were used as positive control.

**Fig. S2.** The transcript levels of immune-related genes (lsATG3, lsATG8, lsATG12) in nonviruliferous treated with ds*LsPellino* were analyzed using RT-qPCR. NS, not significant. *, *p* < 0.05, **, *p* < 0.01 and ***, *p* < 0.001 by the student t-test. The error bars represent the standard error of the mean (SEM). Three independent biological replicates were performed for each experiment.

**Fig. S3. LsPellino participates in other rice virus infection in small brown planthoppers.** (A and B) The expression of LsPellino at the transcript (A) and protein (B) levels in non-infected and RBSDV-infected planthoppers was analyzed using RT-qPCR and Western blotting assays. Each dot represents an insect sample. **, *p* < 0.01 by the student t-test. Three independent replicates (Rep 1, Rep 2 and Rep 3) from non-infected and RBSDV-infected planthoppers were selected for immunoblotting analysis. (C) The protein level of RB-P10 in RBSDV-infected planthoppers treated with ds*GFP*- or ds*LsPellino* was analyzed using Western blotting assay. (D) Interaction between RB-P8 and LsSOCS5 or LsPellino in Y2H assay. Yeast cells co-transformed with AD-T and BD-53 was used as a positive control. Yeast co-transformed with AD-T7 and BD-LsSOCS5 was used as a negative control. (E and F) Interaction between RB-P8 and in GST pull-down assay. The recombinant GST-LsPellino (E) or GST-LsSOCS5 (F) were incubated with GST beads. MBP-RB-P8 was then added to the beads. The bead-bound proteins were analyzed using Western blotting assay. The molecular weight is showed in the left. Arrows indicates the bands of the recombinant proteins.

**Table S1.** Primers used in this study
