# Supplementary material for: A plant virus attenuates the Toll immune pathway by degradation of Pellino to facilitate viral infection in insect vectors
Source: J Virol. 2025 Mar 31;99(5):e00021-25. doi: 10.1128/jvi.00021-25 (PMC12090757; doi:10.1128/jvi.00021-25)
Supplement: Table S1 — Primers used in this study. [file jvi.00021-25-s0005.docx]

**Table S1. Primers used in this study.**

| **Primer name** | **Sequence (5' to 3')** |
| --- | --- |
| RNAi-pellino-F | 5'-TAATACGACTCACTATAGGGAGAGGTGAACGCAGGCCGTCCCG-3' |
| RNAi-pellino-R | 5'-TAATACGACTCACTATAGGGAGACAATTGAATATCAGACGAATG-3' |
| RNAi-SOCS5-F | 5'-GATCACTAATACGACTCACTATAGGGAGACCTGACCCTGTTGTTCCATT-3' |
| RNAi-SOCS5-R | 5'-GATCACTAATACGACTCACTATAGGGAGAGAGTTGACTGACCCCATCG-3' |
| RNAi-GFP-F | 5'-TAATACGACTCACTATAGGCGCGCCGAGGTGAAGTTC-3' |
| RNAi-GFP-R | 5'-TAATACGACTCACTATAGGGTTCACCTTGATGCCGTTC-3' |
| AD-LsPellino-F | 5'-GACGTACCAGATTACGCTCATATGCCAGATGAGAATACAGAC-3' |
| AD-LsPellino-R | 5'-GCAGCTCGAGCTCGATGGATCCTCAATTGAAT ATCAGACGAATGT-3' |
| AD-LsPellino-N-F | 5'-CGACGTACCAGATTACGCTCATATGATGCCAAAGCCCAAACATATATCGG-3' |
| AD-LsPellino-N-R | 5'-ATCTACGATTCATCTGCAGCTCGAGTCATTCTTCTAAATAATCTTTTGTA-3' |
| AD-LsPellino-C-F | 5'-CGACGTACCAGATTACGCTCATATGATGTTGGTAGATAAGGTGAACGCAG-3' |
| AD-LsPellino-C-R | 5'-ATCTACGATTCATCTGCAGCTCGAGTCAATTGAATATCAGACGAATGTAG-3' |
| BD-LsPellino-F | 5'-ATCTCAGAGGAGGACCTGCATATGCCAGATGAGAATACAGAC-3' |
| BD-LsPellino-R | 5'-GCCGCTGCAGGTCGACGGATCCTCAATTGAAT ATCAGACGAATGT-3' |
| AD-SOCS5-F | 5'-GACGTACCAGATTACGCTCATATGATGGGCCAGAGATTAAGTGATC-3' |
| AD-SOCS5-R | 5'-GCAGCTCGAGCTCGATGGATCCTTACTGCTCGTTGTCAAACC -3' |
| AD-SOCS5-N-F | 5'-GACGTACCAGATTACGCTCATATGATGGGCCAGAGATTAAGTGATC-3' |
| AD-SOCS5-N-R | 5'-GCAGCTCGAGCTCGATGGATCCTTAAGGATTGAATTTTGACAAG-3' |
| AD-SOCS5-C-F | 5'-GACGTACCAGATTACGCTCATATGATGGAAGACTATCCAATAGAGGA-3' |
| AD-SOCS5-C-R | 5'-GCAGCTCGAGCTCGATGGATCCTTACTGCTCGTTGTCAAACC-3' |
| BD-SOCS5-C-F | 5'-ATCTCAGAGGAGGACCTGCATATGATGGAAGACTATCCAATAGAGGA-3' |
| BD-SOCS5-C-R | 5'-GCCGCTGCAGGTCGACGGATCCTTACTGCTCGTTGTCAAACC-3' |
| AD-SOCS5-SH2-F | 5'-GACGTACCAGATTACGCTCATATGGTGCCTGATCTGCTGCAAAT-3' |
| AD-SOCS5-SH2-R | 5'-GCAGCTCGAGCTCGATGGATCCTTAAGGCTCAAAAAACATACAGC-3' |
| AD-SOCS5-SOCS-F | 5'-GACGTACCAGATTACGCTCATATGATGCTGACTATCCCGCTG-3' |
| AD-SOCS5-SOCS-R | 5'-GCAGCTCGAGCTCGATGGATCCTTACTGCTCGTTGTCAAACC-3' |
| AD-RSV-NS3-F | 5'-TACGACGTACCAGATTACGCTCATATGatgaacgtgttcacatcgtctgtg-3' |
| AD-RSV-NS33-R | 5'-ATCTACGATTCATCTGCAGCTCGAGctacagcacagctggagagctgcc-3' |
| BD-RSV-NS3-F | 5'-CATATGGCCATGGAGGCCGAATTCatgaacgtgttcacatcgtctgtg-3' |
| BD-RSV-NS3-R | 5'-GCGGCCGCTGCAGGTCGACGGATCCctacagcacagctggagagctgcc-3' |
| AD-RBSDV-P8-F | 5'-TACGACGTACCAGATTACGCTCATATGatgactggcacccatgacgacc-3' |
| AD-RBSDV-P8-R | 5'-ATCTACGATTCATCTGCAGCTCGAGttacacaataatagatgcagcttttag-3' |
| BD-RBSDV-P8-F | 5'-CATATGGCCATGGAGGCCGAATTCatgactggcacccatgacgacc-3' |
| BD-RBSDV-P8-R | 5'-GCGGCCGCTGCAGGTCGACGGATCCttacacaataatagatgcagcttttag-3' |
| Tube-F | 5'-ATGTCTGTAG ATACAGAATTGA-3' |
| Tube-R | 5'-TTATAAAGGT ATTCTTCTTGAGA-3' |
| Myd88-F | 5'-ATGGCAGCTA CTGAGGTGG-3' |
| Myd88-R | 5'-TTAAGCTGGG ACAGGCAGT-3' |
| LsNEDD4-F | 5'-ATGATTTGTGCTCCCAGAACACAAC-3' |
| LsNEDD4-R | 5'-TCAATCAACTCCCGCAAAGCCTTGC-3' |
| LsCYC4-F | 5'-ATGGGGAAACGACAGCATCAAAAGG-3' |
| LsCYC4-R | 5'-TTACCATGAACTGAAATTTCCGAAA-3' |
| LsPARKIN-F | 5'-ATGGCATCAATATTAGAAATAATCT-3' |
| LsPARKIN-R | 5'-TCAGTGACTACGTACGCCACAGCCT-3' |
| LsTRAF6-F | 5'-ATGACTGACATATCTCCGACTGGCA-3' |
| LsTRAF6-R | 5'-TTAACCATTGACACAGCTCAAGCGG-3' |
| LsCUL1-F | 5'-ATGAGCTTGAACAGAGTGATGTCGT-3' |
| LsCUL1-R | 5'-TCACGCAAGATAGCTGTAGGCATCC-3' |
| LsSKP-F | 5'-ATGCCTAGTATCAAGCTGCAGAGTT-3' |
| LsSKP-R | 5'-TCACTTTTCCTCACACCATTCGTTC-3' |
| LsDIAP2-F | 5'-ATGAATTCAGAAGAAAGTAGGTTCT-3' |
| LsDIAP2-R | 5'-TCACGACAGATAGGTGCGGACGGTG-3' |
| GST-LsPellino-F | 5'-TCCAGGGGCCCCTGGGATCCatgCCAAAGCCCAAACATATA-3' |
| GST-LsPellino-R | 5'-GTCAGTCACGATGCGGCCGCTCAATTGAATATCAGACGAATGT-3' |
| Pet28a-Tube-F | 5'-TTTAACTTTAAGAAGGAGATATACCATGTCTGTAGATACAGAATTGAGAA-3' |
| Pet28a-Tube-R | 5'-AGTGGTGGTGGTGGTGGTGCTCGAGTAAAGGTATTCTTCTTGAGATTTCT-3' |
| q-LsPellino-F | 5'-TAACGGATCTTTGCCCCCAG-3' |
| q-LsPellino-R | 5'-ACTCGACGATAATCGCCTGC-3' |
| q-SOCS5-F | 5'-CCGGTAAGTCGTGTCCGAAA -3' |
| q-SOCS5-R | 5'-GCAAACACACGAGTCACACC -3' |
| q-actin-F | 5'-AATCGTAAGAGACATCAAGGAG-3' |
| q-actin-R | 5'-AGGCAATTCGTAGGACTTCT-3' |
| q-RSV-NP-F | 5'-AGGCAATCAATGACATCTCC-3' |
| q-RSV-NP-R | 5'-ATCTCTCACAAAGCCAGTGC-3' |
| q-RB-P10-F | 5'-AACAACCGACCAACAATCAC-3' |
| q-RB-P10-R | 5'-GAGCAGGAACTTCACGACAG-3' |
| q-ATG3-F | 5'-TCACGTGGATCCCACAACTG-3' |
| q-ATG3-R | 5'-TCCTCGTCTAGAAGGCCACT-3' |
| q-ATG8-F | 5'-CGTTATTCCCCCTACCAGCG-3' |
| q-ATG8-R | 5'-TTTCCATAGACGTTCTCATCACT-3' |
| q-ATG12-F | 5'-GTGAAACTGATTTGAATTCAG-3' |
| q-ATG12-R | 5'-GGTTCCTTACTGTCTGGTCAG-3' |
